# Effects of milk fat globule membrane supplementation on physical function and body composition in older adults: a narrative review

**DOI:** 10.1080/15502783.2026.2753931

**Published:** 2026-11-02

**Authors:** Rachel H. Barclay, Marlena C. Kruger, Matthew J. Barnes

**Affiliations:** a School of Sport, Exercise and Nutrition, Massey University, Palmerston North, New Zealand; b School of Health Sciences, Massey University, Palmerston North, New Zealand

**Keywords:** Milk fat globule membrane, phospholipids, healthy aging, dietary supplement, exercise

## Abstract

**Background:**

Healthy aging is a priority for many individuals; however, a decline in physical function and poor body composition are common age-related changes that can cause the added years from rising life expectancy to be spent in poor health. Consequently, understanding the mechanisms involved in aging has become increasingly important, including the role of phospholipids (PLs) and how age-related changes in PL content may interfere with lipid metabolism and normal functioning of the musculoskeletal system. Milk fat globule membrane (MFGM) is a bovine milk fraction rich in PLs that may have the potential to support good physical health, so that quality of life can rise alongside life expectancy.

**Methods:**

This narrative review was based on a literature search conducted on Google Scholar, PubMed, and Scopus in May 2026 using the following keywords and Boolean operators: milk fat globule membrane OR MFGM AND physical function OR body composition OR older adults. Studies were included if they were randomized clinical trials (RCTs) investigating MFGM's effects on physical function and/or body composition in older adults.

**Results:**

Findings suggest that MFGM supplementation, when consumed in conjunction with exercise, is an effective way to improve physical function in older adults, particularly among Japanese cohorts; however, changes in habitual diet in response to exercise, variable PL concentrations, and other added ingredients used in interventions may have influenced these results. In contrast, its effects on body composition appear more modest, with some studies reporting no benefits, especially when exercise was not part of the study design, or when compliance may have been a factor.

**Conclusions:**

Beyond the combined use of supplementation and exercise, future RCTs should consistently report participant compliance and, where possible, examine MFGM as a standalone intervention to distinguish its effects from other bioactive ingredients. Greater standardization is also needed, particularly regarding PL dose. Studies should also aim to include participants from more diverse ethnic backgrounds to capture a wider range of physiological responses and improve generalizability of the findings.

## 1. Introduction

Aging is an inevitable process that is accompanied by numerous age-related structural and functional changes that affect all biological systems, from a cellular level through to the whole organism [1]. This is often associated with a deterioration in physical health that may include reduced physical function and altered body composition, particularly progressive muscle loss or sarcopenia related to age [2,3]. In individuals aged 50 years and older, muscular strength can decline by 1.5 to 5% per year [4–6], and muscle loss can occur at a rate of approximately 1% per year [4]. Additionally, estimates based on longitudinal data suggest that abdominal fat mass can increase yearly by 3 to 5% in men and 5 to 10% in women between the ages of 30 and 70 years [7]. As life expectancy continues to rise globally, increases in lifespan are only favorable when accompanied by good physical health, not prolonged periods of decline [8]. It is also relevant to consider that the physical effects of aging extend beyond the individual, imposing a broader societal and economic burden, most notably on the healthcare system [9]. Consequently, understanding the physical aging process has become increasingly important.

A wide range of theories have been proposed to explain the mechanisms underlying age-related deterioration in physical health, including disruptions in phospholipid (PL) homeostasis [10]. PLs are polar lipid molecules that carry out a variety of roles in the human body and are particularly important for maintaining a well-functioning musculoskeletal system [11]. They support neurotransmitter synthesis, myelin sheath formation (a fatty layer surrounding axons that facilitate the rapid transmission of nerve impulses to the muscle), and the integrity of mitochondrial membranes within skeletal muscle [12]. PLs also contribute to lipid metabolism by transporting cholesterol through the circulatory system and regulating intestinal absorption [13]. With age, there is evidence to suggest that overall PL content changes, despite inconsistent findings as to whether it decreases or increases [1,10]. These alterations may disrupt lipid metabolism and compromise muscle function, with the latter potentially further exacerbated by degenerative age-related changes at the neuromuscular junction (NMJ) [14], thereby contributing to poor physical health in later life.

One nutritional strategy that may offset these common age-related changes and promote healthy aging is consuming milk fat globule membrane (MFGM). MFGM is a membrane that encapsulates triglyceride globules and it is typically derived through the manufacture of dairy products to be used as an ingredient in infant formula, supporting brain growth and development [15]. Its high PL content and reported health benefits in the context of early life nutrition suggest that it is likely to have practical applications in other age groups, including older adults [10,16], especially when it is combined with structured exercise [17], which is a well-established way to slow some of the physical effects of aging [18,19]. While previous reviews have provided excellent summaries on the broader potential health benefits of MFGM [16,20,21], none to date have examined in greater detail those related to physical function and body composition during aging. Therefore, this review article aims to provide a concise narrative review on the current knowledge of MFGM supplementation, specifically its effects on age-related changes in physical function and body composition in older adults.

## 2. Methods

This narrative review was informed by the recommendations proposed in Ferrari [22]. A literature search was conducted on the databases: Google Scholar, PubMed, and Scopus, in May 2026 using the following keywords and Boolean operators: “milk fat globule membrane” OR “MFGM” AND “physical function” OR “body composition” OR “older adults.” Articles were included if they were randomized clinical trials (RCTs) investigating the effects of MFGM supplementation on measures of physical function and/or body composition in older adults (>45 years old) and available to read in English. Articles that did not meet these criteria were excluded. There were no restrictions on publication date, placebo use, presence of other ingredients in the MFGM supplement, or exercise incorporation.

## 3. Age-related changes

### 3.1. Physical function decline

Reductions in physical function can be a defining characteristic of aging [9,23], affecting as many as 37.3% of individuals aged 60 years and over, based on recent estimates [23]. Older adults can experience an annual loss of muscular strength of up to 5%, which may further accelerate over time [6]. This means that over a 10-year period, they can lose up to half of their strength. Additionally, from the age of 70 years, cardiorespiratory fitness has been shown to decline by up to 25% after each passing decade [24]. Decreased balance and mobility may also become more apparent by the age 60 years, as part of declining physical function that often occurs with aging [25].

Good physical function is a core feature of healthy aging [26]. It is crucial for maintaining independence and quality of life well into older age, especially in the context of rising life expectancy [8]. Strength, mobility, and balance are all essential for activities of daily living including walking, standing up from a chair, using the bathroom, and getting dressed. Weakness, immobility, and/or postural instability not only make these tasks more difficult to perform, but they also serve as a strong predictor of future falls and bone fractures [18,27]. For example, decreased leg power and grip strength in older men have been associated with a 19% and 16% greater risk of fall injury, respectively [28]. Following an injurious fall, older adults often face permanent disability, long-term rehabilitation, increased reliance on others for activities of daily living, and, in some cases, death [9,29]. Extending beyond the individual to a broader level, these consequences can place a significant strain on the healthcare system, where reports estimate the total annual cost of fatal and non-fatal falls among older adults to be approximately $50 billion (USD) and rising [30].

While age-related declines in physical function can be primarily attributed to progressive muscle loss [9,31], PLs may also be involved [32]. PLs contribute to normal muscle function by providing phosphatidylcholine (PC) to synthesize acetylcholine, a neurotransmitter that facilitates muscle contraction, phosphatidylethanolamine (PE) to support mitochondrial membrane integrity within skeletal muscle, and sphingomyelin (SM) to myelinate nerves [1,10]. In general, research suggests that the body's overall PL content begins to change around 50 years of age, with PC, PE, PS, and SM levels decreasing with advancing age [1,10,33]. Conversely, other studies report contrasting findings [12,34], with Zarezadeh et al [1]. suggesting that the discrepancies among studies may reflect methodological differences, particularly the failure to stratify PL concentrations by sex and underlying health conditions. Regardless of the direction of change, content alterations may impair the ability of PLs to adequately carry out their force-producing functions within the musculoskeletal system, which is consistent with observed reductions in strength and gait speed among older adults with low plasma lysophosphatidylcholine (a PC derivative) [35]. Therefore, it is plausible that disruptions in PL homeostasis may partly explain age-related declines in physical function beyond those attributable to progressive muscle loss alone.

### 3.2. Altered body composition

#### 3.2.1. Progressive muscle loss

Progressive muscle loss is another hallmark of the aging process [2,36]. Aging has detrimental effects on the musculoskeletal system, which can include a reduction in the number of motor units, fibrosis and stiffening of the muscle extracellular matrix, slowed rates of myofibrillar protein synthesis, and/or myofiber atrophy [18,37–39], particularly that of type II (fast-twitch) fibers [9]. These changes may begin as early as 30 to 40 years of age, with muscle loss occurring gradually over time at an estimated rate of approximately 1% per year, which is usually slower than a decline in physical function [4]. However, beyond 70 years of age, the rate of decline has been shown to accelerate thereafter [39]. Additionally, some evidence suggests that older men experience greater losses than women [39], possibly due to men generally having a higher baseline muscle mass before these losses occur [40].

With the rising number of older people in society, progressive muscle loss is a growing concern. It is both a contributor to and a consequence of declining physical function, creating a cycle whereby muscle loss leads to less function, which in turn accelerates further wastage through decreased activity and disuse [9,41,42]. Subsequently, this can lead to a loss of independence, social isolation, and diminished quality of life [31]. Muscle loss is also associated with increased mortality, as it has been shown to predict both functional decline and an increased likelihood of death [43].

The mechanisms that cause age-related muscle loss involve a combination of physiological and life-style factors, with oxidative damage, deterioration of contractile proteins, decreased satellite cell content, metabolic dysregulation, physical inactivity, and comorbid diseases highlighted as some potential key factors [9,31,44]. Other research implicates reduced PL content, specifically PC and cardiolipin (CL) [45,46], although findings have been inconsistent [34]. PC and CL are highly concentrated within the mitochondria of skeletal muscle, where they help to maintain membrane structure [45]. Consequently, reductions in these PLs have been associated with reduced mitochondrial oxidative capacity and dysfunction [47]. Furthermore, a preclinical trial with mice has demonstrated the potential of dietary PC in restoring mitochondrial function by preserving membrane integrity [48]. However, while these studies may provide evidence to show how reductions in PLs may contribute to age-related muscle loss, the mechanistic link remains unclear, particularly in human studies.

#### 3.2.2. Increase in visceral adiposity

Another characteristic of aging may include an increase in visceral adipose tissue (VAT), where fat accumulates around vital organs in the abdomen, rather than subcutaneous regions [49]. This age-related shift in body composition is supported by lifespan studies, which report a positive correlation between VAT and advancing age in both men and women [50,51]. Data show that between the ages of 30 and 70 years, VAT can increase by over 200% in men and 400% in women [7]; although shown to increase with age, it generally does so until around 65 to 70 years, before declining in later life [52,53]. However, in women, postmenopausal reductions in estrogen may lead to continued increases beyond this age [54].

While it is possible that a certain level of fat mass in older adults may not be entirely detrimental, with studies indicating that being overweight may offer some protection against frailty and early mortality [55], excessive VAT can accelerate the aging process and adversely affect health in numerous ways [56]. It can lead to hypertension, hyperglycemia, and dyslipidaemia [50], increasing the risk of cardiovascular disease, certain cancers, and other obesity-related illnesses [57]. VAT can also promote low-grade systemic inflammation and metabolic dysregulation, which can inhibit muscle protein synthesis and contribute to the development of sarcopenia or a condition known as sarcopenic obesity [58].

Similar to the mechanisms underlying declining physical function and progressive muscle loss, age-related increases in VAT are multifactorial [59], with altered lipid metabolism being a possible contributing factor [60]. Lipid metabolism encompasses processes related to lipid transport, storage, and mobilization, where PLs play important roles, including the transport of cholesterol through the circulatory system and regulation of intestinal lipid absorption [13]. Therefore, changes in PL content with age may affect lipid handling and consequently promote a redistribution of fat toward the abdominal region [60,61]. While research in humans remains limited, an increase in total SM levels in aging mice has been linked to enlarged adipocytes [62], which is consistent with a possible role of PLs in age-related increases in VAT. However, this relationship is not fully understood, particularly as the research is divided on whether overall PL content increases or decreases with age [1,10,12,32–34].

Aging, together with its associated physical and physiological changes, may appear daunting, but exercise is a well-established strategy for maintaining physical function and a healthy body composition into older age [18]. However, barriers such as frailty, pain, fear of injury, and low exercise tolerance may limit participation and adherence [63]. As such, nutritional supplements, including MFGM, have been investigated as a means of enhancing the benefits of even low- to moderate-intensity exercise in this population [38].

## 4. Bovine-derived MFGM

### 4.1. Structure and function

MFGM is commonly extracted from bovine milk during cream processing and primarily marketed for use in infant formula [64]. It is a trilayer membrane of approximately 10–50 nm thick that surrounds triglyceride globules suspended in milk [65]. Within mammary epithelial cells, the inner membrane is formed in the endoplasmic reticulum, while the outer bilayer is formed in the apical plasma membrane [21]. MFGM accounts for 2 to 6% of a triglyceride globule's total mass [20], and functions as a protective barrier that separates the lipid core from the external aqueous environment, which helps to prevent coalescence and hydrolysis [21]. Embedded in MFGM's structure are various bioactive proteins, glycosylated molecules, enzymes, and a range of lipids including cholesterol, free fatty acids, and PLs [66] (see Figure 1). While different components of MFGM may exert therapeutic potential, PLs are of particular importance given their diverse functions within the human body [67,68].

**Figure 1.**
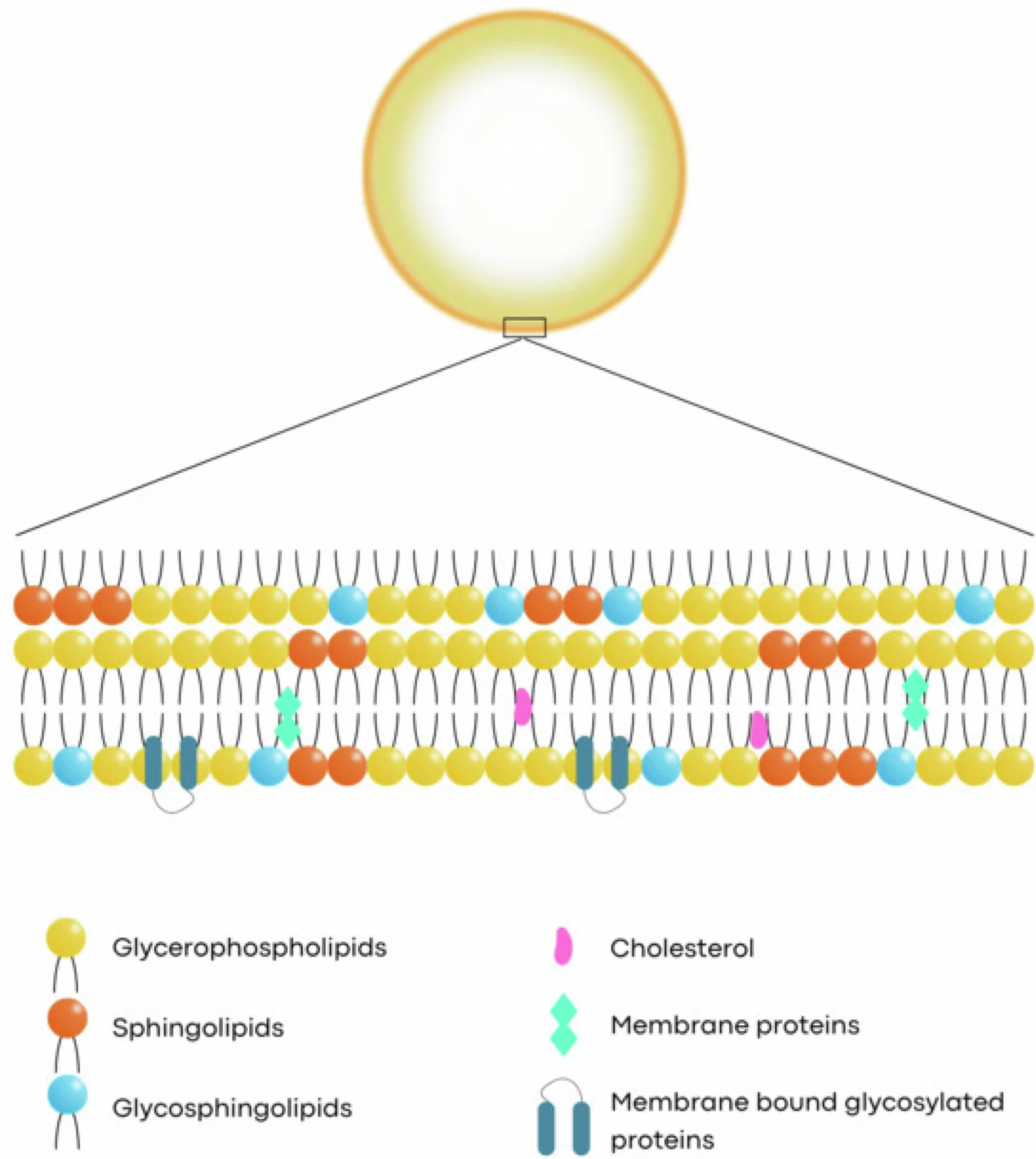
Diagram of the milk fat globule membrane. Reproduced from: Slykerman R, Davies *N*, Fuad M, Dekker J. Milk fat globule membranes for mental health across the lifespan. Foods. 2024; 13(11):1631. Licensed under CC BY.

### 4.2. The MFGM PLs

PLs are polar lipid molecules with phosphate-containing heads and fatty acid tails that self-assemble to form the basic structure of MFGM, where they constitute up to 15 to 30% of its total composition [20]. Of the many PL species documented [68], those primarily found in MFGM are PC, PE, phosphatidylserine (PS), phosphatidylinositol (PI), and SM [69]. While the concentration of these primary PLs can vary based on isolation and processing methods, stage of lactation, cow breed, and other factors, PC and PE are the most abundant, constituting approximately 26.1% and 22.9%, respectively, according to findings from profiling of lipids in bovine milk [70]. Following these, SM is also present in relatively high concentrations and accounts for around 19% of the total PLs in MFGM [20]. Although the underlying mechanisms are not fully understood, it is possible that the PLs in MFGM may benefit the physical health of older adults by supplementing the body's PL content to support key PL-mediated functions that are important for a well-functioning neuromuscular system, such as PE for mitochondrial membrane stability [12] and SM for the myelination of nerves [64].

### 4.3. Evidence from RCTs

#### 4.3.1. Effects on physical function

Recent RCTs have mostly reported beneficial effects of MFGM on physical function in older adults [17,38,64,71–78], particularly when it is consumed alongside exercise. Daily supplementation with 1 g/day of MFGM tablets (333 mg PLs) combined with a weekly exercise routine has been shown to improve strength, mobility, gait speed, and reaction time in older Japanese adults, as demonstrated by increased isometric knee extension strength [38], and shorter completion times for the timed-up-and-go test [73]. In a comparable population sample from Japan, participants who received a similar daily dose of MFGM, but with a lower PL content (184 mg), and performed cardiovascular exercise twice a week had significantly higher side-step scores compared to those that received a placebo [76]. The intervention group also demonstrated improved neuromuscular functioning during knee extension [76], which corresponds to findings from similar studies [38,78].

Aging is associated with deterioration of the NMJ, which is characterized by fragmentation, loss of structural integrity, and denervation [79]. Findings from preclinical studies suggest that MFGM combined with exercise improves motor function by attenuating age-related morphological changes in the NMJ in mice [14,80]. These effects have been attributed, in part, to the SM content of MFGM, which may upregulate the expression of genes involved in NMJ formation and maintenance [80]. SM may also support the myelination of nerves, which could indirectly preserve NMJ integrity [14]. However, direct structural evidence of this occurring in humans is currently lacking, with RCTs largely inferring NMJ adaptations from improvements to broader physical outcomes. Therefore, further research is needed to determine whether these findings translate from mice to humans, particularly given the likely differences in MFGM dosages.

The evidence from RCTs to date suggests that MFGM supplementation is an effective nutritional strategy to support physical function in later life, particularly when it is accompanied with exercise training [17,38,64,72,73,76,78], as presented in Table 1. While changes in habitual diet could have been a confounding factor, particularly if protein or energy intake was modified following the introduction of structured exercise, this was generally accounted for through the use of diet records [17,38,64,71,72,76,78], with only one study reporting a significant between-group difference in protein intake [72]. Regarding the MFGM intervention, although many studies administered a similar dose (e.g. 1 g/day), the PL content varied greatly between studies, likely reflecting differences in extraction and/or processing methods [70]. As PLs are the proposed bioactive ingredient [68], it is important to consider whether this variability may have had a substantial influence on the findings. Additionally, as research has predominantly focused on Japanese cohorts, it is uncertain whether performance benefits extend to other populations, because race and ethnicity have been shown to influence physiological responses to exercise due to genetic, lifestyle, and environmental differences [81].

**Table 1.** Effects of MFGM supplementation on physical function in older adults.

Author	Study design	Participants	Exercise intervention	Nutritional intervention	Duration	Physical function measures	Key findings	Compliance
Daly et al. [17]	Randomized, double-blind, placebo-controlled, 2-arm parallel-group study	Sedentary females from Australia (*n* = 216 (MFMD:108, placebo:108), 45–65 y)	Resistance exercise incl. balance and mobility (2×/week in the gym and 1×/week at home (added at week-5))	30 g × 2/day of MFMD from cow's milk that contained 200 mg of MFGM-derived PLs with other nutrients (e.g. vitamin D) or 30 g x2/day of rice powder blend placebo	4 months	5- and 10-step stair climb, sit-to-stand, squat jump, CMJ, 1RM leg press, isometric dorsiflexion, grip strength, 4 m walk test, timed-up-and-go, stepping reaction time, single-leg stand (eyes open and closed), sit and reach, 2-min step test	5-step regular-pace stair descent time (*p* = 0.045), CMJ height (*p* = 0.038), single leg balance eyes closed (*p* = 0.005), 2-min step (*p* = 0.003), sit and reach performance (*p* = 0.026) improved in MFMD group	Overall compliance: 78% for exercise and 91% for nutritional intervention. Neither differed between groups
Jyväkorpi et al [72].	Randomized, parallel, 2-arm control-group study	Home-dwelling women from Finland (*n* = 94 (MFGM: 44, control: 50), ≥ 70 y)	A short five-movement body weight exercise routine everyday	Intervention group had a choice between one of two protein-containing snack products: 250 ml/day chocolate milk shake with 3.6 g MFGM (1.2 g PLs) or 30 g/day of protein powder with 3.6 g MFGM (1.3 g PLs), or control	12 weeks	5-time chair stand, walking speed, balance (all measured separately, as well as collectively in SPPB test), grip strength	MFGM improved balance (*p* = 0.022) and SPPB test score (*p* = 0.020) from baseline to 12 weeks	92.1% for the snack product. 81.% (MFGM) vs 70.7% (control) for exercise, with a difference between groups (*p* = 0.015)
Kim et al. [73]	Randomized, double-blind, placebo-controlled, 4-arm parallel-group study	Frail females from Japan (*n* = 126 (ex+MFGM: 33, ex+placebo: 31, MFGM: 30, placebo: 32), > 75 y)	1 hr resistance exercise class incl. balance and gait training using body weight and resistance bands (2×/week)	1 g/day of MFGM (333 mg PLs) or 1 g/day of whole milk powder placebo (2.86 mg PLs)	12 weeks	Grip strength, isometric knee extension, 5 m walking speed, timed-up-and-go	Ex+MFGM increased usual walking speed compared to MFGM alone (*p* = 0.005). Ex+MFGM also improved timed-up-and-go performance compared to all other groups (*p* = 0.005)	Authors did not report exercise or nutritional compliance
Kim et al. [74]	Randomized, double-blind, placebo-controlled, 4-arm parallel-group study	Community-dwelling Japanese females with declines in walking ability (*n* = 122 (ex+MFGM: 29, ex+placebo: 31, MFGM: 31, placebo: 31), > 79 y)	1 hr resistance exercise class incl. balance and gait training using body weight and resistance bands (2×/week)	1 g/day of MFGM (333 mg PLs) or 1 g/day of whole milk powder placebo (2.86 mg PLs)	12 weeks	Grip strength, isometric knee extension, 5 m walking speed, timed-up-and-go	Usual walking speed increased in both exercise groups compared to MFGM and placebo alone groups (*p* = 0.001)	Authors did not report exercise or nutritional compliance
Kokai et al. [75]	Randomized, double-blind, placebo-controlled, 2-arm parallel-group study	Community-dwelling Japanese adults (*n* = 113 (MFGM: 57, placebo: 56), 50–70 y)	No exercise	1 g/day of MFGM (38 mg PLs) or 1 g/day of whole milk powder placebo (1.3 mg PLs)	24 weeks	Isometric knee extension, 5-time chair stand, chair stepping, single-leg stand (eyes closed)	Single-leg stand time increased in MFGM group from baseline to 24 weeks (*p* = 0.046)	Authors did not report nutritional compliance
Minegishi et al [38].	Randomized, double-blind, placebo-controlled, 2-arm parallel-group study	Healthy Japanese adults (*n* = 22 (MFGM: 11, placebo: 11), 60–73 y)	15 min of low-intensity walking and 15 min of cycle training (2x/week)	1 g/day of MFGM (333 mg PLs) or 1 g/day of whole milk powder placebo (2.86 mg PLs)	10 weeks	Isometric knee extension, 5-time chair stand, EMG during knee extension	MFGM increased isometric strength (*p* < 0.05) and muscle fiber conduction velocity (*p* < 0.05) from baseline to 10 weeks	Overall compliance for intervention protocol was > 98%
Ota et al. [76]	Randomized, double-blind, placebo-controlled, 2-arm parallel-group study	Healthy Japanese adults (*n* = 44 (MFGM: 19, placebo: 16), 50–69 y)	15 min of walking and 15 min of cycle training at moderate intensity (2×/week)	1 g/day of MFGM (184 mg PLs) or 1 g/day of whole milk powder placebo (3.22 mg PLs)	10 weeks	Isometric and isokinetic knee extension, side-step, 5-time chair stand, EMG during knee extension	Side-step performance increased (*p* = 0.047) and muscle fiber conduction velocity improved (*p* = 0.046) from baseline to 10 weeks in MFGM group	Overall compliance for intervention protocol was > 98%
Suzukamo et al [77].	Randomized, double-blind, placebo-controlled, 2-arm parallel-group study	Healthy Japanese adults (*n* = 53 (MFGM: 25, placebo: 28), 60-74 y)	No exercise	1 g/day of MFGM (38 mg PLs) +1.5 g of glucosamine or isoenergetic placebo/day (type and amount not specified)	8 weeks	ROM (knee flexion and back rotation), 10 m walking speed, 10 m obstacle walking, open-close stepping, single-leg stand (eyes open and closed), timed stair climb	Knee flexion ROM (*p* = 0.031), 10 m obstacle walking speed (*p* = 0.045), and speed of ascending stair (*p* = 0.036) increased in MFGM+glucosamine group	Nutritional compliance: 99.8% (MFGM) vs 99.6% (placebo)
Watanabe et al [78].	Randomized, double-blind, placebo-controlled, 2-arm parallel-group study	Healthy Japanese adults (*n* = 24 (MFGM: 12, placebo: 12), 63–84 y)	Isometric knee extension training at > 80% of MVC (2×/week)	1 g/day of MFGM (43.6 mg PLs) or 1 g/day of whole milk powder placebo (no PLs detected)	8 weeks	EMG during isometric knee extension	MFGM improved motor unit firing rates at 2, 4, 6, and 8 weeks (*p* = < 0.05)	Authors did not report exercise or nutritional compliance
Yoshinaka et al [64].	Randomized, double-blind, placebo-controlled, 2-arm parallel-group study	Healthy Japanese adults (*n* = 62 (MFGM: 32, placebo: 30), ~75 y)	Light rhythmic exercise (moving multiple body parts in parallel for 120 beats for 1 min) performed at home daily	1 g/day of MFGM (160 mg PLs) or 1 g/day of whole milk powder placebo (3.22 mg)	8 weeks	10 m walking speed, isometric knee extension, foot tapping, open-close stepping test	Foot tapping and open-close stepping scores were significantly higher from baseline to 8 weeks in MFGM group (*p* < 0.05)	Authors did not report exercise or nutritional compliance
Zajac et al. [71]	Randomized, double-blind, placebo-controlled, 3-arm parallel-group study	Healthy adults with self-reported memory complaints from Australia (*n* = 222 (LD: 75, HD: 71, placebo: 76), 55–75 y)	No exercise	50 g/day of powdered milk fortified with LD MFGM (1.7 g PLs) or HD MFGM (4 g PLs), or 50 g/day of rice starch placebo	16 weeks	Grip strength	The grip strength of males in HD MFGM group improved but only in participants aged 55–64 y (*p* < 0.05)	Authors did not report nutritional compliance

*Note. Multi-nutrient-fortified milk drink (MFMD), milk fat globule membrane (MFGM), phospholipids (PLs), countermovement jump (CMJ), one-repetition maximum (1RM), short physical performance battery (SPPB), exercise group (ex), electromyography (EMG), range of motion (ROM), maximal voluntary contraction (MVC), low-dose (LD), high-dose (HD). Intervention, statistical findings, and compliance are reported as described by the authors. n = number of participants that completed the study.Effects on Body Composition.*

RCTs have also explored the effects of MFGM supplementation on body composition in older adults [71,73,82], with several incorporating structured exercise into their study design [38,64,74,78]. Positive changes in body composition occurred when sedentary females consumed MFGM-derived PLs in a daily multi-nutrient fortified milk drink (MFMD) and participated in resistance exercise, with increased lean muscle mass, greater muscle cross-sectional area, and reduced adiposity observed after 4 months [17]. While it is possible that MFGM was responsible for these changes, it is important to note that the MFMD contained added protein, calcium, and vitamin D, which may have had a greater impact, particularly as MFGM received less attention in the authors' discussion [17]. Conversely, studies where the intervention was not fortified with other bioactive ingredients have attributed positive changes in body composition specifically to the effects of MFGM [76], with Kim et al. [74] suggesting that it promotes skeletal muscle hypertrophy by supporting pathways involved in muscle protein synthesis. This could explain the findings of Minegishi et al. [38], who reported improvements in muscle cross-sectional area following 10 weeks of daily MFGM supplementation and cardiovascular exercise in adults over 60 years compared to a placebo.

MFGM may also improve body composition through pathways involved in lipid metabolism [68]. It has been suggested that the cholesterol-binding properties of SM may modify blood lipid profiles, which may contribute to reductions in fat mass [20,65]. A meta-analysis on six RCTs, involving 464 participants, found that MFGM supplementation significantly reduced total cholesterol and low-density lipoprotein levels, possibly due to inhibiting lipid absorption in the gut [13]. Another mechanism could be its suppressive effects on adipose tissue. A pre-clinical trial on mice fed a high-fat diet found that MFGM supplementation blocked preadipocytes from maturing by altering pathways involved in their differentiation, which attenuated increases in body weight [83]. However, although several RCTs have investigated MFGM and body composition, relatively few have examined the underlying mechanisms by which it may influence lipid regulation and possibly reduce fat mass.

Despite some positive changes in body composition being reported, to date most studies have not observed any benefits following MFGM supplementation [64,74,75,78], as presented in Table 2. In a 14-week study primarily investigating cognitive health, older adults experienced no significant changes in body composition after consuming 200 ml/day of an MFGM-enriched milk drink (~377 mg PLs) [82]. Similarly, 1 g/day of MFGM tablets was not enough to improve bioelectrical impedance analysis (BIA), a common method used to assess body composition, after 24 weeks in older Japanese adults [75]. Neither study assigned their participants a structured exercise program, and as MFGM is proposed to enhance the effects of exercise [38], supplementation alone may have not been enough to stimulate any beneficial changes in body composition. Alternatively, in studies that did incorporate exercise, but observed no benefit, it is possible that participants did not fully adhere to the study protocol; however, it is difficult to determine the influence of compliance when it is not consistently reported [64,73,74,78].

The evidence supporting improved body composition following MFGM supplementation in older adults is less consistent than that for physical function, with fewer studies observing improvements in measures of muscle mass and fat mass. It appears that, without an exercise intervention, MFGM supplementation alone may not be enough to improve body composition. This suggests that structured exercise, particularly of sufficient intensity to minimize the exercise dose-response effect, should be performed concomitantly. Conversely, where exercise was included in the study design, poor adherence may have reduced the effectiveness of supplementation. Lastly, the independent effects of MFGM from those of other added ingredients with ergogenic potential, such as glucosamine [77] or added protein [72], are difficult to distinguish.

**Table 2.** Effects of MFGM supplementation on body composition in older adults.

Author	Study design	Participants	Exercise intervention	Nutritional intervention	Duration	Body composition measures	Key findings	Compliance
Calvo et al. [82]	Randomized, double-blind, placebo-controlled, 2-arm parallel-group study	Healthy or mildly cognitively impaired adults from Spain (*n* = 40 (MFGM: 19, placebo: 21), > 65 y)	No exercise	200 ml/day of a MFGM-enriched milk drink (~377 mg PLs) or 200 ml/day of an UHT skim milk drink placebo	14 weeks	BIA, WC	No significant differences in body composition between groups (total FM: *p* = 0.4407, total MM: *p* = 0.8668, WC: *p* = 0.4383)	Authors did not report compliance for nutritional intervention
Daly et al. [17]	Randomized, double-blind, placebo-controlled, 2-arm parallel-group study	Sedentary females from Australia (*n* = 216 (ex+MFMD:108, ex+placebo:108), 45–65 y)	Resistance exercise incl. balance and mobility (2×/week in the gym and 1×/week at home (added at week-5))	30 g ×2/day of MFMD from cow's milk that contained 200 mg of MFGM-derived PLs with other nutrients (e.g. vitamin D) or 30 g ×2/day of rice powder blend placebo	4 months	DXA, muscle CSA and density	Body composition improved in MFMD group, with decreased total FM (*p* = 0.004), total fat % (*p* < 0.001), leg FM (*p* = 0.037), and trunk FM (*p* < 0.002), and increased total LM (*p* = 0.020) and muscle CSA (thigh: *p* = 0.003; lower leg: *p* = 0.005)	Overall compliance: 78% for exercise and 91% for nutritional intervention. Both did not differ between groups
Kim et al. [73]	Randomized, double-blind, placebo-controlled, 4-arm parallel-group study	Frail females from Japan (*n* = 126 (ex+MFGM: 33, ex+placebo: 31, MFGM: 30, placebo: 32), > 75 y)	1 hr resistance exercise class incl. balance and gait training using body weight and resistance bands (2×/week)	1 g/day of MFGM (333 mg PLs) or 1 g/day of whole milk powder placebo (2.86 mg PLs)	12 weeks	Frailty criterion (incl. weight loss), DXA	At follow up, ex+MFGM group had a significantly greater reversal of the weight loss frailty criterion compared to all other groups (*p* = 0.005)	Authors did not report compliance for exercise or nutritional interventions
Kim et al. [74]	Randomized, double-blind, placebo-controlled, 4-am parallel-group study	Community-dwelling Japanese females with declines in walking ability (*n* = 122, (ex+MFGM: 29, ex+placebo: 31, MFGM: 31, placebo: 31), > 79 y)	1 hr resistance exercise class incl. balance and gait training using body weight and resistance bands (2×/week)	1 g/day of MFGM (333 m**g** PLs) or 1 g/day of whole milk powder placebo (2.86 mg PLs)	12 weeks	DXA	No significant differences in body composition between groups after 12 weeks (arm MM: *p* = 0.274, leg MM: *p* = 0.051, skeletal MM: *p* = 0.075)	Authors did not report compliance for exercise or nutritional interventions
Kokai et al. [75]	Randomized, double-blind, placebo-controlled, 2-arm parallel-group study	Community-dwelling Japanese adults (*n* = 113, (MFGM: 57, placebo: 56), 50-70 y)	No exercise	1 g/day of MFGM (38 mg PLs) or 1 g/day of whole milk powder placebo (1.3 mg PLs)	24 weeks	MRI of CSA	No change in CSA after 24 weeks of MFGM compared to a placebo (*p* = 0.28)	Authors did not report compliance for nutritional intervention
Minegishi et al [38].	Randomized, double-blind, placebo-controlled, 2 -arm parallel-group study	Healthy Japanese adults (*n* = 22 (MFGM: 11, placebo: 11), 60–73 y)	15 min of low-intensity walking and 15 min of cycle training (2×/week)	1 g/day of MFGM (333 mg PLs) or 1 g/day of whole milk powder placebo (2.86 mg PLs)	10 weeks	BIA, MRI of CSA	CSA increased from baseline to week 10 in MFGM group (*p* < 0.05)	Overall compliance for intervention protocol was > 98%
Ota et al. [76]	Randomized, double-blind, placebo-controlled, 2-arm parallel-group study	Healthy Japanese adults (*n* = 44 (MFGM: 19, placebo: 16), 50–69 y)	15 min of walking and 15 min of cycle training at moderate intensity (2×/week)	1 g/day of MFGM (184 mg PLs) or 1 g/day of whole milk powder placebo (3.22 mg PLs)	10 weeks	MRI of CSA	MFGM group had significantly greater muscle CSA from baseline to 10 weeks compared to placebo group (*p* = 0.025)	Overall compliance for intervention protocol was > 98%
Watanabe et al [78].	Randomized, double-blind, placebo-controlled, 2-arm parallel-group study	Healthy Japanese adults (*n* = 24, 63–84 y)	Isometric knee extension training at > 80% of MVC (2x/week)	1 g/day of MFGM (43.6 mg PLs) or 1 g/day of whole milk powder placebo (no PLs detected)	8 weeks	BIA, quadricep thickness	No significant between-group differences in body composition (total and lower limb MM at week 4 and 8: *p* = 0.728) or muscle thickness (week 2, 4, 6, and 8: *p* > 0.05)	Authors did not report exercise or nutritional compliance
Yoshinaka et al [64].	Randomized, double-blind, placebo-controlled, 2-arm parallel-group study	Healthy Japanese adults (*n* = 62 (MFGM: 32, placebo: 30), ~75 y)	Light rhythmic exercise (moving multiple body parts in parallel for 120 beats for 1 min) performed at home daily	1 g/day of MFGM (160 mg PLs) or 1 g/day of whole milk powder placebo (3.22 mg)	8 weeks	BIA	No significant inter- or intragroup differences in body composition were found (*p-*value not reported)	Authors did not report compliance for exercise or nutritional interventions
Zajac et al. [71]	Randomized, double-blind, placebo-controlled, 3-arm parallel-group study	Healthy adults with self-reported memory complaints from Australia (*n* = 222 (LD: 75, HD: 71, placebo: 76), aged 55–75 y)	No exercise	50 g/day of powdered milk fortified with LD MFGM (1.7 g PLs) or HD MFGM (4 g of PLS), or 50 g/day of rice starch placebo	16 weeks	BIA	Placebo group had significant increases in total FM (vs. LD: *p* = 0.024, HD: *p* = 0.001) and fat %, (vs. LD: *p* = 0.036, HD: *p* = 0.006) and decreases in fat-free % (vs. LD: *p* = 0.036, HD: *p* = 0.006) and skeletal MM % (vs. LD: *p* = 0.031, HD: *p* = 0.008)	Authors did not report nutritional compliance

*Note. Milk fat globule membrane (MFGM), phospholipids (PLs), bioimpedance analysis (BIA), waist circumference (WC), multi-nutrient-fortified milk drink (MFMD), dual-energy X-ray absorptiometry (DXA), cross-sectional area (CSA), fat mass (FM), muscle mass (MM), lean mass (LM), muscle cross-sectional area (CSA), exercise (ex), magnetic resonance imaging (MRI), maximal voluntary contraction (MVC), low-dose (LD), high-dose (HD). Intervention, statistical findings, and compliance are reported as described by the authors. n = number of participants that completed the study.Safety and Tolerance of MFGM Supplementation.*

Earlier applications of MFGM in research involving infants and children have demonstrated good safety profiles of MFGM-enriched formulas [84,85]. Within RCTs investigating its effects on physical function and body composition in older adults, MFGM is similarly well tolerated, with no adverse side effects related to supplementation being reported by most studies [38,64,71–77,82], except for one trial [17]. While minor, Daly et al [17]. found that participants in the intervention group reported a higher incidence of bloating, diarrhea, and other gastrointestinal symptoms than the placebo group (MFMD: 22 vs. placebo: 8), which resulted in four participants (3.2% of those participating overall) discontinuing supplementation. These adverse effects were attributed to possible undiagnosed lactose intolerance or malabsorption, due to the MFMD being dairy-based; however, as it was tolerated by most participants, the authors still considered it safe for consumption [17].

## 5. Conclusion

A range of physical changes occur across the lifespan as part of the natural aging process. It is important to recognize that increases in life expectancy are not inherently beneficial if the added years are spent in poor health. Many older adults maintain good physical health well into old age; however, others experience declines in physical function and adverse changes in body composition, which have far-reaching consequences not only for the individual but also for society. While altered PL content may contribute to these common outcomes of advancing age, the mechanisms through which they occur are yet to be fully established, particularly in human research. MFGM may help minimize the impact of aging by improving physical function, especially when it is combined with exercise, which is itself one of the most effective and well-established strategies for healthy aging. However, the potential for MFGM supplementation to improve body composition in older adults is not as widely supported, and more research is needed in this area, particularly studies that incorporate structured exercise into the study design.

Alongside the incorporation of exercise, future studies should account for potential confounding factors to improve the attribution of effects to MFGM, including habitual dietary intake and participant compliance, the latter of which should be consistently monitored and reported. There is also a need for greater participant diversity and standardization of MFGM interventions, specifically PL content and additional fortification. In turn, this may facilitate more comparable and generalizable findings, expanding current knowledge of MFGM supplementation and its effects on age-related changes in physical function and body composition in older adults.
